# Acid Sphingomyelinase Inhibitor, Imipramine, Reduces Hippocampal Neuronal Death after Traumatic Brain Injury

**DOI:** 10.3390/ijms232314749

**Published:** 2022-11-25

**Authors:** Si Hyun Lee, A Ra Kho, Song Hee Lee, Dae Ki Hong, Beom Seok Kang, Min Kyu Park, Chang Juhn Lee, Hyun Wook Yang, Seo Young Woo, Se Wan Park, Dong Yeon Kim, Bo Young Choi, Sang Won Suh

**Affiliations:** 1 Department of Physiology, College of Medicine, Hallym University, Chuncheon 24252, Republic of Korea; 2 Neuroregeneration and Stem Cell Programs, Institute for Cell Engineering, School of Medicine, Johns Hopkins University, Baltimore, MD 21205, USA; 3 Department of Neurology, School of Medicine, Johns Hopkins University, Baltimore, MD 21205, USA; 4 Department of Physical Education, Hallym University, Chuncheon 24252, Republic of Korea; 5 Institute of Sport Science, Hallym University, Chuncheon 24252, Republic of Korea

**Keywords:** traumatic brain injury, acid sphingomyelinase, ceramide, imipramine, neuronal death

## Abstract

Traumatic brain injury (TBI) broadly degrades the normal function of the brain after a bump, blow, or jolt to the head. TBI leads to the aggravation of pre-existing brain dysfunction and promotes neurotoxic cascades that involve processes such as oxidative stress, loss of dendritic arborization, and zinc accumulation. Acid sphingomyelinase (ASMase) is an enzyme that hydrolyzes sphingomyelin to ceramide in cells. Under normal conditions, ceramide plays an important role in various physiological functions, such as differentiation and apoptosis. However, under pathological conditions, excessive ceramide production is toxic and activates the neuronal-death pathway. Therefore, we hypothesized that the inhibition of ASMase activity by imipramine would reduce ceramide formation and thus prevent TBI-induced neuronal death. To test our hypothesis, an ASMase inhibitor, imipramine (10 mg/kg, i.p.), was administrated to rats immediately after TBI. Based on the results of this study, we confirmed that imipramine significantly reduced ceramide formation, dendritic loss, oxidative stress, and neuronal death in the TBI-imipramine group compared with the TBI-vehicle group. Additionally, we validated that imipramine prevented TBI-induced cognitive dysfunction and the modified neurological severity score. Consequently, we suggest that ASMase inhibition may be a promising therapeutic strategy to reduce hippocampal neuronal death after TBI.

## 1. Introduction

Traumatic brain injury (TBI) is common, leading to cognitive dysfunction and neuronal death. In particular, the number of young adult TBI patients has increased due to motor-vehicle collisions and falls [1,2,3]. In the United States, the number of TBI patients is approximately 1.6 million, and more than 50,000 deaths result from TBI each year [1]. When the brain suffers an impact, intracranial hematomas form and the brain swells. Subsequently, secondary damage, such as inflammatory reactions, blood–brain barrier destruction, and oxidative stress, occur [4,5]. Secondary injuries are especially dangerous for patients because they lead to neuroexcitotoxicity, cerebral metabolic dysfunction, inflammation, neuronal death, and even cognitive dysfunction [3,6,7,8].

Sphingomyelin is a lipid and constructs plasma membrane leaflets in mammalian cells [9]. Sphingomyelinase (SMase) is an enzyme that hydrolyzes sphingomyelin to ceramide on the leaflets of plasma membranes. The two isoforms of SMase are distinguished by pH optima [10,11]: one is neutral sphingomyelinase (NSMase) and the other is acidic sphingomyelinase (ASMase) [12]. ASMase performs an essential housekeeping function and maintains sphingolipid homeostasis by converting sphingolipid to ceramide in lysosomes [13]. However, under pathologic conditions, ASMase is overactivated and then induces excessive ceramide production in cells [14,15,16,17]. Sphingomyelin turnover induces the release of apoptotic factors, such as Fas ligand (Fas-L) and tumor necrosis factor (TNF)-α, which causes the rapid accumulation of sphingolipids [18]. In this respect, ASMase performs an important function in cell survival and cell membrane composition and is an essential enzyme for homeostasis.

Ceramide consists of a fatty acid and sphingosine, which acts as a bioactive molecule that regulates multiple cellular pathways, including the cell cycle, stress response, proliferation, infections, neurodegeneration, and apoptosis [9,19,20]. In its normal state, ceramide supports the structure of membranes and mediates cellular functions, including responses to external stimuli, proliferation, migration, and death [21,22]. However, under pathological conditions, the role of ceramide changes to activating the neuronal apoptotic pathways by causing inflammation, secreting apoptotic cytokines, or producing excessive ROS [18,23,24]. Inordinate ceramide formation leads to proapoptosis in neurons, and the sulfatide species in ceramides, such as C18:0 and C24:1, are abundant in neurons, astrocytes, and myelin [25]. Therefore, the balance of ceramide is important, and related studies are ongoing. However, the association between ASMase/ceramide and neuronal death in TBI remains unclear.

Tricyclic antidepressants (TCAs) have three ring structures and have been used as depression treatments by inhibiting norepinephrine and serotonin reuptake [26,27]. In addition, they have a cationic group that acts as an ASMase inhibitor by competing with the ASMase in the plasma membrane [23,28]. One of these TCAs, imipramine, has a treatment effect on psychiatric diseases and reduces the activity of ASM to below 50% in cells by attaching to the lysosomal inner membrane [29,30]. Furthermore, imipramine is used to protect neurons from inflammation and apoptosis [31,32]. Imipramine has a neuroprotective effect via the inhibition of ASMase in transient global ischemia and hypoglycemia [33,34,35].

In this study, we investigated whether imipramine, as an ASMase inhibitor, can reduce ceramide formation and, thus, prevent TBI-induced neuronal death in rats. To verify our hypothesis, we immediately administrated imipramine (10 mg/kg. i.p.) after TBI. We measured ASMase and ceramide activation at 3 h after TBI. We evaluated ASMase expression, ceramide abundance, oxidative stress, dendritic loss, and glial cell activations by immunofluorescence assays 24 h after TBI. In addition, we performed the mNSS test every days for 7 days after the TBI, and then we sacrificed animals to detect live neurons by NeuN. We also performed a Morris water maze (MWM) test for 5 days from the 8th to 12th day after the TBI. We suggest the therapeutic potential of imipramine in hippocampal neuronal death and cognitive dysfunction after TBI.

## 2. Results

### 2.1. Imipramine Reduces Acid Sphingomyelinase and Ceramide Overexpression after Traumatic Brain Injury

**Figure 1:**
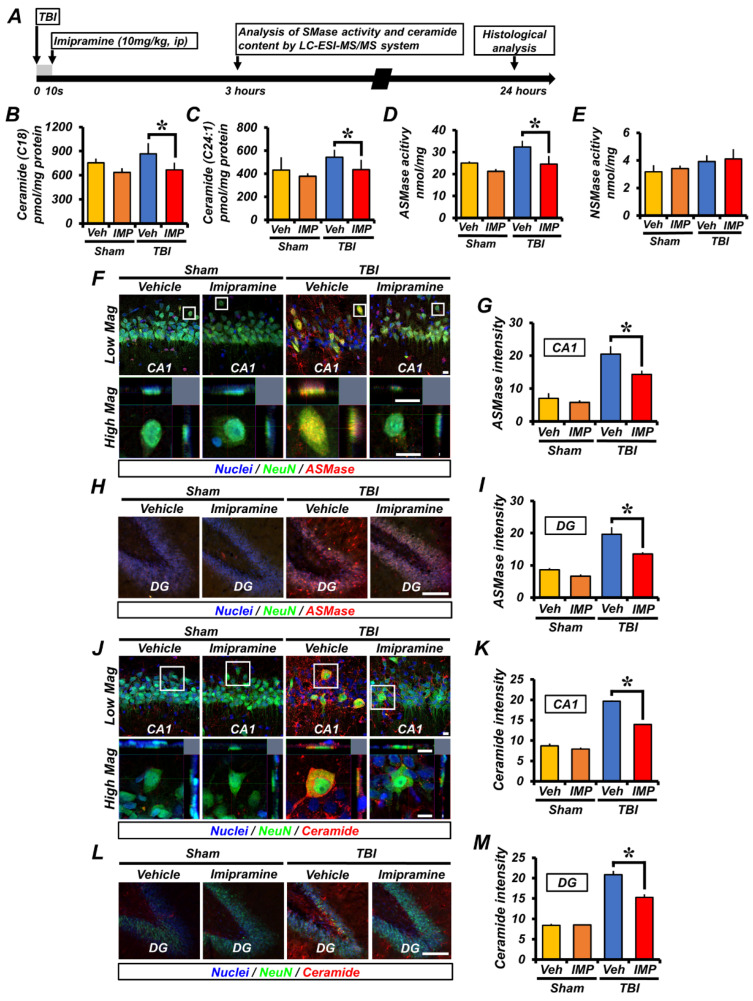
Imipramine treatment reduced ASMase and ceramide activity after TBI. (**A**) Experimental procedures are demonstrated by timeline. Imipramine was injected immediately after TBI impact. Next, ASMase and ceramide analyses were performed 3 h after TBI. Histological analysis was performed 24 h after TBI. (**B**,**C**) Quantification of ceramide (C18 and C24:1) activity. (**D**,**E**) Quantification of ASMase and NSMase. Data are mean ± SEM; n = 3 from each sham group, n = 7 from TBI-vehicle group, and n = 8 from TBI-imipramine group. (**F**,**H**) Fluorescent images show effect of imipramine on ASMase activity. ASMase (red) intensity shown for hippocampal CA1 and DG in sham-operated, TBI-vehicle, and TBI-imipramine groups. Scale bar = 10 and 100 µm, respectively. (**G**,**I**) Quantification of ASMase fluorescence intensity on hippocampal CA1 and DG. Data are mean ± SEM; n = 4 from each sham group; n = 6 from each TBI group. * *p* < 0.05 vs. the vehicle-treated TBI group. (**J**,**L**) Fluorescent images show effect of imipramine on ceramide activity. Ceramide (red) intensity shown on hippocampal CA1 and DG in sham-operated, TBI-vehicle, and TBI-imipramine treated groups. Scale bar = 10 and 100 µm, respectively. (**K**,**M**) Quantification of ceramide fluorescence intensity on hippocampal CA1 and DG. Data are mean ± SEM; n = 4 from each sham group; n = 6 from each TBI group. * *p* < 0.05 vs. vehicle-treated TBI group.

We first measured the levels of ceramide subtypes, C18 (sham-vehicle, 755.423 ± 52.38; sham-imipramine, 634.71 ± 54.58; TBI-vehicle, 865.92 ± 134.3; TBI-imipramine, 665.98 ± 91.26, a 23.1% decrease) and C24:1 (Sham-vehicle, 431.26 ± 111.26; sham-imipramine, 377.907 ± 26.03; TBI-vehicle, 542.47 ± 64.98; TBI-imipramine, 435.04 ± 84.77, a 19.2% decrease), 3 h after TBI. Compared with the sham-vehicle group, the TBI-vehicle group had a higher level of ceramide subunits. However, the levels of both types of ceramides were significantly reduced by administration of imipramine (Figure 1B,C). We also measured the activities of ASMase and NSMase. The level of ASMase activity also increased after TBI compared with that in the sham groups. However, imipramine treatment significantly reduced the levels of ASMase activity after TBI (sham-vehicle, 25.01 ± 0.76; sham-imipramine, 21.2 ± 1.08; TBI-vehicle, 32.37 ± 2.79; TBI-imipramine, 24.56 ± 3.71, a 24.13% decrease). In addition, there were no differences for the NSMases activity between groups (sham-vehicle, 3.17 ± 0.49; sham-imipramine, 3.41 ± 0.21; TBI-vehicle, 3.92 ± 0.45; TBI-imipramine, 4.1 ± 0.72). This means that imipramine influenced only the ASMase level (Figure 1D,E).

Next, we performed ASMase immunofluorescence staining to confirm excessive ASMase production 24 h after TBI. In the sham-operated groups, the ASMase expression level was no different between the vehicle and imipramine-treated groups. However, the ASMase expression in the TBI-vehicle group was higher than in the sham-operated groups. The administration of imipramine reduced the TBI-induced ASMase overexpression. According to the results in Figure 1, imipramine treatment dramatically reduced the ASMase fluorescence signal in the hippocampal CA1 (sham-vehicle, 7.02 ± 1.55; sham-imipramine, 5.82 ± 0.63; TBI-vehicle, 542.47 ± 64.98; TBI-vehicle, 20.5 ± 1.4; TBI-imipramine, 14.3 ± 0.7, a 30.2% decrease) and DG (sham-vehicle, 8.67 ± 0.48; sham-imipramine, 6.64 ± 0.54; TBI-vehicle, 19.6 ± 1.3; TBI-imipramine, 13.5 ± 0.3, a 31.1% decrease) regions (Figure 1F–I). We also performed immunofluorescence staining with an antibody against ceramide to analyze the ceramide levels of the neurons. As with the ASMase expression results, the sham-operated groups showed no significant difference in ceramide levels. In the TBI-vehicle group, we confirmed that excessive ceramide was generated owing to the increased overexpression of ASMase after injury. Additionally, we confirmed that treatment with imipramine significantly decreased the production of ceramide via the inhibition of ASMase in the CA1 (sham-vehicle, 8.7 ± 0.99; sham-imipramine, 7.89 ± 0.65; TBI-vehicle, 19.7 ± 0.4; TBI-imipramine, 13.9 ± 0.1, a 29.4% decrease) and DG (sham-vehicle, 8.3 ± 0.77; sham-imipramine, 8.5 ± 0.23; TBI-vehicle, 20.8 ± 0.9; TBI-imipramine, 15.3 ± 0.8, a 26.4% decrease) compared with that in the TBI-vehicle group (Figure 1J–M). These results suggested that imipramine reduces ceramide overexpression by inhibiting ASMase activation in the hippocampal CA1 and DG after TBI. These results indicated that imipramine can reduce ceramide levels by specifically inhibiting ASMase after TBI.

### 2.2. Imipramine Reduces TBI-Induced Hippocampal Neuron Death

**Figure 2:**
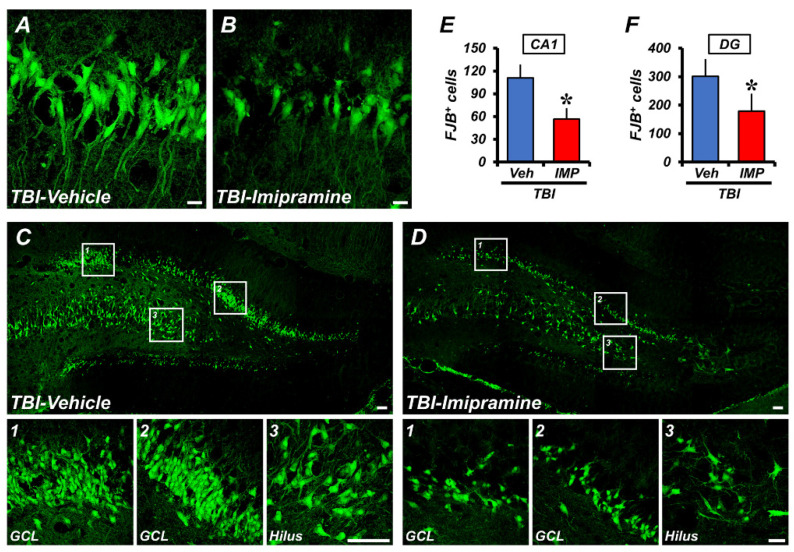
Imipramine treatment reduces hippocampal neuron death 24 h after TBI. (**A**,**B**) Representative fluorescence images showing degenerating neurons stained with Fluoro-Jade B (FJB) in hippocampal CA1 regions after TBI. Scale bar = 10 µm. (**C**,**D**) Representative fluorescence images showing degenerating neurons stained with FJB in hippocampal DG (granular cell layer and hilus) regions after TBI. Scale bar = 50 µm. (**E**,**F**) Quantification of number of degenerating neurons in TBI-vehicle and TBI-imipramine groups. Data are mean ± SEM; n = 6 from each TBI group. * *p* < 0.05 vs. vehicle-treated TBI group.

To investigate the effect of imipramine, we evaluated TBI-induced hippocampal neuronal death by using Fluoro-Jade B (FJB) staining. We immediately injected imipramine (10 mg/kg) and harvested the brains 24 h after TBI. We performed the FJB staining on the harvested brain to evaluate the number of degenerating neurons in the TBI-vehicle and TBI-imipramine groups. After comparing the TBI-vehicle and TBI-imipramine groups, we found that imipramine treatment considerably reduced the number of degenerating neurons. Specifically, the imipramine significantly reduced the level of degenerating neurons in the CA1 (TBI-vehicle, 110.8 ± 17.7; TBI-imipramine, 56.6 ± 14.5, a 48.9% decrease) and DG (TBI-vehicle, 301.5 ± 24.5; TBI-imipramine, 179.1 ± 40.8, a 40.6% decrease) regions (Figure 2A,B). These results indicated that imipramine treatment can reduce hippocampal neuronal death after TBI.

### 2.3. Imipramine Reduces Oxidative Damage and Dendritic Loss after TBI

**Figure 3:**
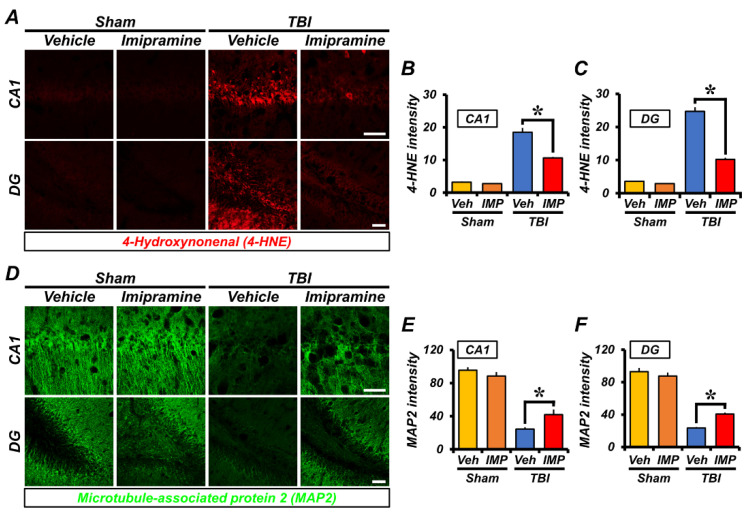
Imipramine treatment decreased lipid peroxidation and microtubule damage 24 h after TBI. (**A**) Immunofluorescence images showing lipid peroxidation marker 4-HN-stained (red) hippocampal CA1 and DG. Scale bar = 100 µm. (**B**,**C**) Quantification of 4-HNE fluorescence-positive intensity in hippocampal CA1 and DG. Data are mean ± SEM; n = 4 from each sham group; n = 6 from each TBI group. * *p* < 0.05 vs. vehicle-treated TBI group. (**D**) Representative fluorescence images showing microtubule marker MAP-2-stained (green) hippocampal CA1 and DG with nuclei (blue). Scale bar = 100 µm. (**E**,**F**) Quantification of MAP-2 intensity in hippocampal CA1 and DG. Data are mean ± SEM; n = 4 from each sham group; n = 6 from each TBI group. * *p* < 0.05 vs. vehicle-treated TBI group.

To compare the levels of oxidative stress in the sham-operated, TBI-vehicle, and TBI-imipramine groups at 24 h, we performed 4-hydroxynonenal (4HNE) immunofluorescence staining to detect lipid peroxidation, which is an indirect marker of oxidative damage. As a result, we found no significant difference in oxidative-stress signals between the sham-vehicle and sham-imipramine groups. However, in the TBI-vehicle group, the oxidative signals were stronger in hippocampal CA1 and DG. The imipramine-treatment group had a lesser degree of oxidative stress than the TBI-vehicle group in the hippocampal CA1 (sham-vehicle, 3.2 ± 0.55; sham-imipramine, 2.78 ± 0.13; TBI-vehicle, 18.7 ± 1.1; TBI-imipramine, 10.6 ± 0.3, a 43.3% decrease) and DG (Sham-vehicle, 3.59 ± 0.6; sham-imipramine, 2.89 ± 0.4; TBI-vehicle, 25 ± 1.7; TBI-imipramine, 10.2 ± 0.5, a 59.7% decrease) regions (Figure 3A–C). This means that ROS and oxidative stress were induced by TBI, especially in the CA1 and DG regions, but treatment with imipramine reduced the oxidative stress in the hippocampal CA1 and DG.

Next, to reveal whether imipramine treatment could protect neurons from TBI-induced axonal damage, we used microtubule-associated protein 2 (MAP-2) staining, which detects microtubules in neurons 24 h after TBI. As a result, the sham-operated group showed intact microtubules in the whole brain in the vehicle and imipramine-treated groups. However, the TBI-vehicle group experienced significant microtubule damage compared with that in the sham-vehicle group. In the TBI-imipramine group, the TBI-induced disruption of microtubules was repaired. Specifically, these results displayed a decrease in dendritic loss in the hippocampal CA1 of the TBI-imipramine group (sham-vehicle, 96.57 ± 3.41; sham-imipramine, 88.5 ± 4.76; TBI-vehicle, 24.6 ± 0.9; TBI-imipramine, 41.8 ± 2.6, a 41.1% decrease) and DG (sham-vehicle, 92.82 ± 6.71; sham-imipramine, 87.60 ± 4.04; TBI-vehicle, 23.4 ± 0.5; TBI-imipramine, 40.6 ± 2.0, a 42.4% decrease; Figure 3D–F). These results showed that imipramine can reduce dendritic loss after TBI.

### 2.4. Imipramine Reduces TBI-Induced Astrocyte and Microglia Activation

**Figure 4:**
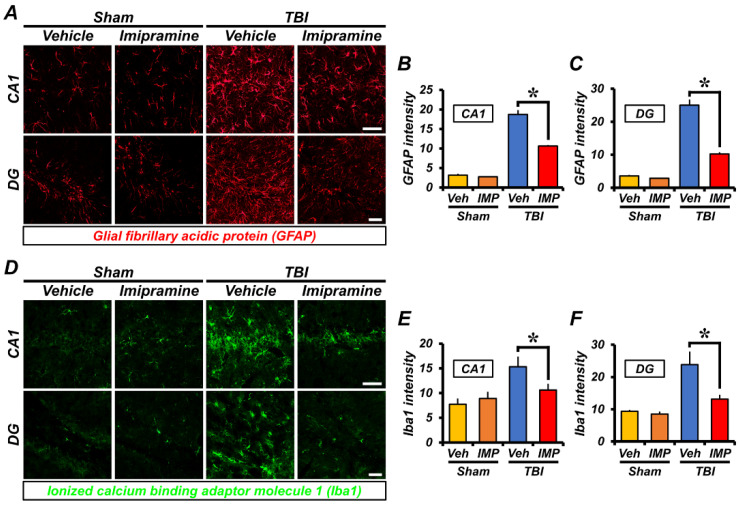
Imipramine treatment reduced glial activation 24 h after TBI. (**A**) Representative images showing astrocytes and microglia stained with GFAP (red) in the hippocampal CA1 and DG. Scale bar = 100 µm. (**B**,**C**) Quantification of astrocyte activation in hippocampal CA1 and DG. Data are mean ± SEM; n = 4 from each sham group; n = 6 from each TBI group. * *p* < 0.05 vs. vehicle-treated TBI group. (**D**) Immunofluorescence images showing Iba-1 (green) in hippocampal CA1 and DG. Scale bar = 100 µm. (**E**,**F**) Bar graph represents activation intensity of microglia in hippocampal CA1 and DG. Data are mean ± SEM; n = 4 from each sham group; n = 6 from each TBI group. * *p* < 0.05 vs. vehicle-treated TBI group.

To quantify excessive inflammation, we next analyzed the microglia and astrocyte activation 24 h after TBI. We performed double immunofluorescence staining to monitor the microglia and astrocytes by ionized calcium-binding adapter molecule 1 (Iba-1) and glial fibrillary acidic protein (GFAP). First, the GFAP intensity was similar in the sham-vehicle and sham-imipramine groups. The rats in the TBI-vehicle group had a higher level of astrocyte activation than those in the sham-vehicle group. In addition, the TBI-vehicle group had a strong fluorescence signal for GFAP compared with that in the TBI-imipramine group due to insult. This suggests that the astrocyte activation increased. However, the GFAP intensity was lower in the TBI-imipramine group in the hippocampal CA1 (sham-vehicle, 11.9 ± 2.7; sham-imipramine, 10.21 ± 2.02; TBI-vehicle, 30 ± 0.6; TBI-imipramine, 20.7 ± 0.8, a 31% decrease) and DG (sham-vehicle, 16.92 ± 5.19; sham-imipramine, 16.25 ± 2.38; TBI-vehicle, 29.7 ± 1; TBI-imipramine, 20.7 ± 1.1, a 30.3% decrease) regions (Figure 4A–C).

The signal of Iba-1 activation was weak, and we observed no difference in the signals between the sham-vehicle and imipramine group. In the TBI-imipramine group, the microglia activation was dramatically reduced in the hippocampal CA1 (sham-vehicle, 7.72 ± 1.18; sham-imipramine, 8.89 ± 1.34; TBI-vehicle, 15.3 ± 0.8; TBI-imipramine, 10.6 ± 0.5, a 30.7% decrease) and DG (sham-vehicle, 9.33 ± 0.49; sham-imipramine, 8.52 ± 0.76; TBI-vehicle, 23.8 ± 1.6; TBI-imipramine, 13.1 ± 0.6, a 45% decrease) compared with that in the TBI-vehicle group (Figure 4D–F). These results indirectly indicated that the inhibition of ASMase can reduce excessive inflammation in the hippocampus.

### 2.5. Imipramine Reduced TBI-Induced Neuronal Death and Cognitive Impairment

TBI causes not only physical damage to the brain, but also impairment of neurological and cognitive function [36]. To test whether imipramine could reduce TBI-induced neuronal death and the extent of cognitive disorders, we performed neuronal nuclei (NeuN) staining, the modified neurological severity score (mNSS) test, and the Morris water maze (MWM) after TBI.

**Figure 5:**
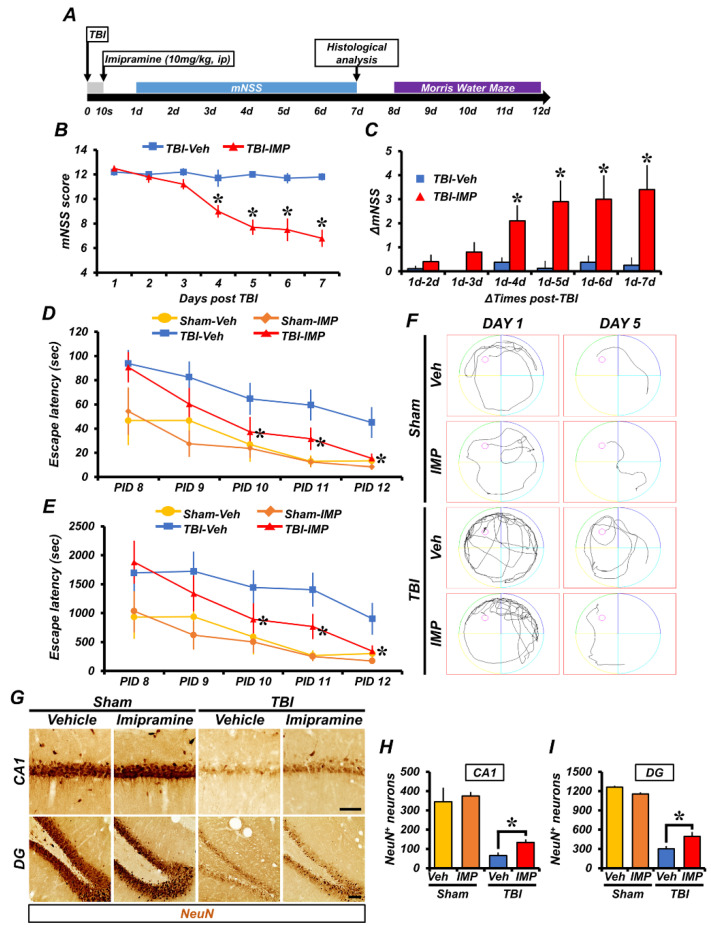
Imipramine restored TBI-induced delayed neuronal loss, neurological deficits, and memory dysfunction. (**A**) Experimental procedures are demonstrated by timeline. We performed the mNSS test every day for 7 days and then histological analysis. We performed the Morris water maze (MWM) test for five consecutive days from day 8 to 12 after TBI. (**B**) mNSS determined in TBI-operated groups on days 1–7 after TBI. Score of up to 18 means that all tasks failed; score of 0 means that all tasks succeeded. (**C**) delta-mNSS determined in TBI-operated groups. Data are mean ± SEM; n = 4 from each sham group; n = 6 from each TBI group. (**D**) We performed the Morris water maze (MWM) test on days 8–12 after TBI. We recorded the platform arrival time for 5 consecutive days. (**E**) Distance taken to arrive at platform for same schedule on 5 consecutive days of MWM. (**F**) MWM tracking record of sham-operated and TBI-operated groups at start and termination of MWM. Data are mean ± SEM; n = 5 from each sham group; n = 10 from each TBI group. (**G**) Representative images showing live neurons detected by NeuN in hippocampal CA1 and DG regions 1 week after TBI in sham-operated groups. Scale bar = 100 µm. (**H**,**I**) Quantification of number of live neurons in hippocampal CA1 and DG 1 week after TBI. Data are mean ± SEM; n = 4 from each sham group; n = 6 from each TBI group. * *p* < 0.05 vs. vehicle-treated TBI group.

We first performed NeuN staining to confirm the live neurons 7 days after TBI. We injected imipramine once, immediately after the TBI, and we harvested the brains on the seventh day after the TBI. In the sham-operated groups, we found no significant difference between the vehicle and imipramine-treated groups, both of which had large amounts of live neurons. However, in the TBI-vehicle group, the number of live neurons was remarkably decreased due to insult compared with the sham-vehicle group. The number of live neurons in the TBI-imipramine group was dramatically increased in the hippocampal CA1 (sham-vehicle, 345.03 ± 145.79; sham-imipramine, 374.83 ± 42.52; TBI-vehicle, 65.64 ± 16.8; TBI-imipramine, 133.34 ± 16.4, a 50.8% decrease) and DG (sham-vehicle, 1262.2 ± 39.91; sham-imipramine, 1154.1 ± 50.91; TBI-vehicle, 267.8 ± 35.9; TBI-imipramine, 447.46 ± 57.1, a 40% decrease) regions (Figure 5G–I). These results indicated that imipramine treatment can reduce hippocampal neuronal death after TBI.

For the mNSS, we performed a test on the neurological disorder severity test every day for 7 days after the TBI. The TBI-imipramine group showed a reduction in the mNSS score compared with the TBI-vehicle group. Specifically, from the fourth to seventh day, the TBI-imipramine group had a significantly reduced score compared with the TBI-vehicle group. In addition, we measured the delta-mNSS, that is, the reduction rate of the neurological disorder score compared with the first day; it also decreased in the imipramine-treated group (Figure 5B,C). In other cases, we also performed a Morris water maze (MWM) test to evaluate the cognitive impairment for 5 days from day 8 to 12 after the TBI. We measured the MWM escape latency and MWM distance to reach the target platform. As shown in Figure 5, the sham-operated group showed that short escape time and distance were associated with a lack of damage in the hippocampus and related to normal cognitive functioning. However, for the TBI-vehicle group, we recorded long escape times and distances, suggesting failure to find the platform. Imipramine treatment showed an effect on finding the platform compared with the TBI-vehicle group. The tracking record showed that the rats in the sham-operated group easily found the platform, whereas those in the TBI groups failed to find the platform. However, on the last day, the TBI-imipramine group found the platform, as with the sham groups. These results showed that imipramine has an effect on recovery from cognitive dysfunction and neurological disorder after TBI (Figure 5D–F).

## 3. Discussion

**Figure 6:**
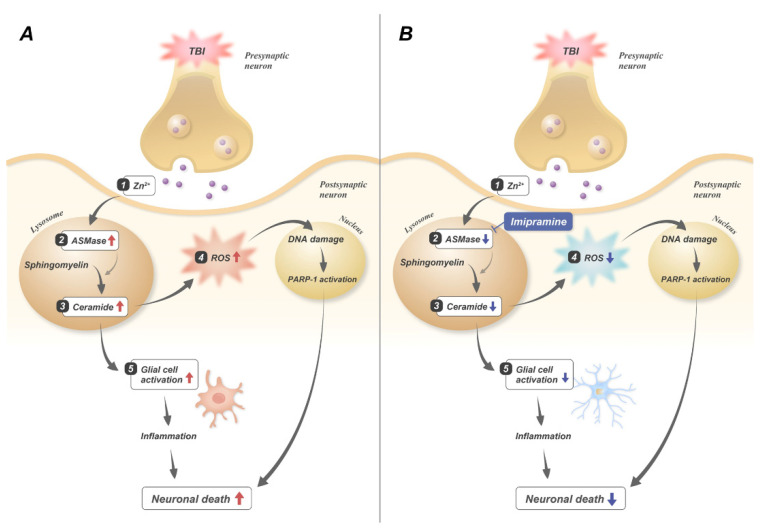
Hypothetical connections between imipramine and TBI-induced neuronal death. (**A**) TBI-induced neuronal death mechanism; (1) under TBI condition, excessive zinc relation and translocation occur; (2) excessive zinc induces overactivation of ASMase; (3) abnormal ASMase activation increases excessive ceramide production; (4) excessive ceramide increases ROS production; (5) increased ceramide induces glial activation. Finally, neuronal death abnormally occurs in hippocampus. (**B**) Imipramine treatment reduces neuronal death by inhibiting ASMase in lysosomes. Imipramine decreases ASMase activation and ceramide formation, after which ROS and glial cell activation are also reduced. Therefore, neuronal death decreases in hippocampus after TBI.

In this study, we investigated whether imipramine administration reduces traumatic-brain-injury (TBI)-induced neuronal death and cognitive dysfunction via the inhibition of acid sphingomyelinase (ASMase). TBI-induced neuronal death is caused by zinc accumulation, oxidative stress, excitotoxicity, and inflammation [37,38,39]. The results of our previous studies have demonstrated that TBI increased the release of vesicular zinc from the presynaptic terminals [35,40,41]. Thus, we think that presynaptically released zinc can activate ASMase and then increase ceramide production, which later induces a neuron death cascade after TBI. ASMase has zinc-binding motifs that are directly activated by zinc [42]. In addition, ASMase and ceramide levels are also highly elevated, resulting in mitochondrial dysfunction and apoptosis [43,44,45]. However, the functional inhibitors of acid sphingomyelinase detach ASMase from the lysosomal inner membrane and degrade it [46]. Therefore, we hypothesized that one of the ASMase inhibitors, imipramine, may reduce TBI-induced neuronal death and cognitive dysfunction. Imipramine inhibits ASMase by reuptake of monoamines such as norepinephrine, dopamine, and serotonin. Additionally, imipramine has anti-inflammatory and antiapoptotic effects [47,48]. In this study, we found that imipramine administration reduced the number of degenerating neurons, oxidative damage, dendritic loss, glial activation, and cognitive dysfunction (Figure 6). Thus, the inhibition of ASMase may be a therapeutic target for TBI-induced hippocampal neuronal death.

We focused on targeting ASMase, which is an enzyme that hydrolyzes ASM to ceramide. In general, ASMase repairs damage to injured neurons, and ceramide regulates cellular proliferation, differentiation, and apoptosis by activating cell damage cascades [49,50]. However, under pathologic conditions, ASMase and ceramide levels are abnormally increased and induce neuronal death. In this study, we used imipramine, which attached to the lysosomal membrane instead of ASMase [31,46]. ASMase is most activated 2–3 h after injury [51,52]. Therefore, we confirmed ASMase activity levels at 3 h after TBI. We found that the activation of ASMase and the formation of ceramide were significantly decreased in the imipramine-treated TBI group compared with those in the vehicle-treated TBI group. This suggested that imipramine acted in acidic conditions and was degraded by ASMase by detaching from the lysosomal membrane. The levels of ceramide isoforms, especially C18 and C24:1, were also decreased in the hippocampus because these subunits constitute most of the ceramide [37]. This suggested that because the production of ceramide was reduced because of ASMase inhibition, the main subunits that consisted of ceramide may have been reduced. These results demonstrated that imipramine administration can reduce ASMase activation and ceramide formation.

Reactive-oxygen-species (ROS)-mediated oxidative stress exacerbates TBI-induced neuronal damage [40,53,54]. Ceramide induces ROS production, which, in turn, stimulates ROS/TNF-α-ceramide cycling [55,56]. In this study, we found that imipramine administration decreased hippocampal oxidative stress in the rats compared with in the TBI-vehicle group. This suggested that ceramide-induced oxidative stress was reduced via the inhibition of ASMase activation. Our results demonstrated that the inhibition of ASMase activation caused reductions in oxidative stress and neuronal death after TBI.

TBI affects neuronal circuitry by destroying the connections between neurons, affecting dendrites and axons [57]. In addition, our previous findings demonstrated that TBI leads to microtubule loss and oxidative stress in the hippocampus [53]. Furthermore, the ASMase–ceramide system is associated with supporting axons and the loss of actin-based microvilli membrane structures [58,59,60]. Therefore, in this study, we confirmed the microtubule disruptions via MAP2 staining to test whether imipramine administration could reduce TBI-induced microtubule damage. We found that imipramine administration reduced ceramide-induced dendritic loss. This result suggested that the suppression of ASMase activation reduced ceramide formation and the subsequent microtubule disruption.

Glial cells perform a neuroprotective role by removing damaged cells and preparing for remodeling after injury [39]. TBI-induced ROS stimulate the release of proinflammatory cytokines, such as TNF-α, IL-6, and IL-10, which are related to glial activation and axonal dysfunction [8,39]. In addition, the activation of the ASMase–ceramide system induces proinflammatory cytokines, such as the release of TNF-α [61]. Sphingolipid metabolism alteration leads to the dysregulation of glial cell activation and inflammatory-mediator synthesis [62]. Therefore, we confirmed the astrocyte and microglial activation via GFAP and Iba1 staining. We found that imipramine administration reduced glial cell activation. The production of ceramide-induced ROS was decreased by the inhibition of ASMase, and the stimulation of the release of proinflammatory cytokines was also reduced. Thus, the activation of glial cells was reduced by the suppression of ASMase. These results suggested that imipramine can protect neuronal cells from TBI-induced inflammation by inhibiting ASMase.

TBI-induced neuronal death results in cognitive impairment [63]. Specifically, in the hippocampus, which is associated with learning and memory, the activation of neurotrophic factors is altered by brain injury [64]. Therefore, we performed neural nuclei staining and a behavior test, and tested neurological deficits and spatial perception by the mNSS for 1 week after TBI; in addition, we conducted a Morris water maze test for 5 days from day 8 to 12 after TBI. According to the results, imipramine administration prevented delayed neuronal loss, neurological deficits, and learning and memory dysfunction. Furthermore, treatment with imipramine preferentially reduced apoptosis caused by oxidative stress, dendritic loss, and inflammation, which subsequently improved cognitive ability. Thus, we suggest that imipramine can reduce neuronal death and cognitive impairment.

Sphingomyelin and ceramide are important in regulating physiological homeostasis involving the cell cycle, proliferation, and stress responses to external stimuli. However, TBI activates neuroinflammation, apoptotic cell damage, and oxidative stress by rapidly accumulating ceramide through activated ASMase. Our findings demonstrated that the inhibition of lysosomal ASMase by imipramine reduced TBI-induced oxidative damage, dendritic loss, glial cell activation, and cognitive impairment. Taken together, the results suggest that the reduction in ceramide concentrations through ASMase inhibition under pathological conditions may offer a potential therapeutic approach to decreasing TBI-induced neurological deficit.

## 4. Materials and Methods

### 4.1. Ethics Statement and Experimental Animals

This animal experiment was approved in accordance with the Laboratory Animal Rules and Laboratory Animal Guides. This study met the criteria of the Experimental Animal Research Committee (Protocol # Hallym 2020-11). We used adult male SD rats (Sprague-Dawley rats, 300–350 g, aged 8 weeks, DBL Co., Eumseong, Republic of Korea) in this study. We allowed all experimental animals to adapt for 1 week under conditions of sustained humidity (55 ± 5%) and temperature (22 ± 2 °C). Room lighting was automatically managed to maintain a 12 h light/dark cycle every day (at 6 a.m.−6 p.m.).

### 4.2. Traumatic BI Surgery

**Figure 7:**
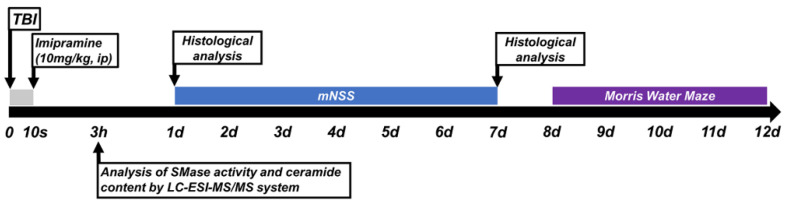
Experimental procedures are demonstrated by timeline. Imipramine was injected immediately after TBI impact. ASMase and ceramide analyses were performed at 3 h after TBI. Histological analyses (ASMase, ceramide, FJB, 4HNE, MAP2, Iba1, and GFAP) were performed at 24 h after TBI. The mNSS test was performed every day for 7 days. Histological analysis for detecting live neurons (NeuN) was performed at 7 days after TBI. The Morris water maze (MWM) test was performed for five consecutive days from day 8 to day 12 after TBI.

We used an electromagnetic cortical impact device to perform TBI surgery. We positioned the rats in a stereotaxic apparatus and deeply anesthetized them with 1–1.5% isoflurane and a 70:30 mixture of nitrous oxide:oxygen (David Kopf Instruments, Tujunga, CA, USA). We fixed the rats by ear bar and performed craniotomy (2.8 mm Lambda from the midline and 3.0 mm lateral from the midline). With a portable drill, we formed a 3 mm diameter hole over the hemisphere. The 3.0 mm flap tip impactor hit the brain at a 5 m/s velocity and reached 3.0 mm in depth. To avoid shivering following hypothermia, we consistently maintained the animal’s body temperature at 36–37.5 °C using a heating pad. We immediately intraperitoneally injected imipramine (10 mg/kg, dissolved in 0.9% normal saline, once) after termination of TBI [35,65,66,67] (Figure 7).

### 4.3. Brain Sample Preparation

We deeply anesthetized the rats with urethane (1.5g/kg, intraperitoneally) in 0.9% normal saline. After anesthetization, we perfused the rats with 0.9% saline and then 4% paraformaldehyde (PFA) in phosphate-buffered saline (PBS) for tissue fixation. We immediately removed the brains after perfusion, which we postfixed for about 1 h with 4% PFA. After postfixation, we moved the obtained brains to 30% sucrose for cryoprotection. After the brain sank in the 30% sucrose solution, we used a cryostat to slice the brain to a thickness of 30 μm, and we stored sectioned brain slices at 4 °C.

### 4.4. Evaluation of Hippocampal Neuronal Death

To confirm hippocampal neuronal death 24 h after TBI, we used Fluoro-Jade-B (FJB) staining to detect degeneration of neurons. Each TBI group comprised 6 rats. We placed the sliced brains on gelatin-coated slides, which we then dried in a dry oven. With 0.06% potassium permanganate and 0.001% FJB solution (Histo-Chem Inc., Jefferson, AR, USA), we performed immunofluorescence staining of the brains of the TBI-vehicle and TBI-imipramine treatment groups. We soaked the slides in xylene for 2 min, which we then covered with cover slides and DPX (Sigma-Aldrich Co., St Louis, MO, USA). After FJB staining, we observed the brain sections with a fluorescence microscope (Olympus, Tokyo, Japan). We quantified the FJB-positive neurons in hippocampal CA1 and DG regions with a 450–490 nm excitation light.

### 4.5. Evaluation of ASMase and Ceramide

We performed an enzyme activity assay to measure ASMase and ceramide 3 h after TBI with an LC-ESI-MS/MS system. We perfused the animals with cold saline, and we harvested the brains 3 h after TBI. Each sham group comprised 3 rats, the TBI-vehicle group comprised 7, and the TBI-imipramine group comprised 8 rats. After brain tissue lysis, with 100 µg, we performed lipid extraction for ASMase and ceramide analysis. Next, we analyzed ceramide and sphingomyelinase contents with LC-MS/MS. We performed ASMase and ceramide immunofluorescence staining to analyze the effect of imipramine 24 h after TBI. With the sectioned brain tissues, we performed ASMase and ceramide double staining. We washed the sectioned brains with 0.01% phosphate-buffered saline (PBS) 3 times for 10 min. Next, with rabbit anti-ASMase (diluted 1:100, Invitrogen, Grand Island, NY, USA) and mouse anti-ceramide (diluted 1:10, Enzo Life Science, Enzo Biochem, Inc., Farmingdale, NY, USA) primary antibodies, we performed immunofluorescence staining. Next, we mounted the stained brains with DPX, which we then covered with cover slides. We observed the ASMase and ceramide with a fluorescence microscope.

### 4.6. Immunofluorescence Assay

To evaluate the effect of imipramine after TBI, we performed immunofluorescence 24 h after TBI [68]. Each sham and TBI group comprised 4 and 6 rats, respectively. We washed the brain tissues with 0.01% PBS 3 times for 10 min. Next, we soaked the brain tissues for 15 min in 1.2% hydrogen peroxide at room temperature to block intracellular peroxidase. We washed the brain tissues again for 10 min 3 times with 0.01% PBS. Next, we immersed the tissues in PBS solution containing Triton X-100 with the primary antibodies and kept overnight. In this study, we used 4-HNE (diluted 1:500; Alpha Diagnostic Intl. Inc., San Antonio, TX, USA), Iba1 (diluted 1:500; Abcam, Cambridge, UK), GFAP (diluted 1:1000, Abcam, Cambridge, UK), and MAP2 (diluted 1:200; Abcam) primary antibodies. Next, we washed the tissues with 0.01% PBS, after which we stained the secondary antibodies, 4-HNE and GFAP, with Alexa-Fluor-594 conjugated antibody, and we stained MAP2 and Iba1 with Alexa-Fluor-488 conjugated antibody (diluted 1:250, Invitrogen, Grand Island, NY, USA) with 4,6-diamidino-2-phenylindole (DAPI; Invitrogen, Grand Island, NY, USA) fluorescence staining for 2 h. Next, we mounted the stained brains with DPX, which we observed with a fluorescence microscope. Subsequently, we analyzed the results with Image J to measure the oxidative stress, microtubules, astrocytes, and microglia.

### 4.7. Detection of Live Neurons

To confirm the neuroprotective effect of imipramine after TBI, we performed a NeuN immunohistochemistry assay 7 days after TBI. Each sham and TBI group comprised 4 and 6 rats, respectively. We harvested the brains and sectioned them in 30 µm slices. After washing with 0.01% PBS buffer, we immersed the brain tissues in mouse anti-NeuN primary antibodies (diluted 1:500, Millipore, Billerica, MA, USA) and PBS solution containing Triton X-100. Next, we used secondary antibody, anti-mouse IgG (diluted 1:250; Vector, Burlingame, CA, USA), for immunohistochemistry assay. With the ABC solution (Vector, Burlingame, CA, USA) and 3,3′ diaminobenzidine (DAB, Sigma-Aldrich Co., St. Louis, Mo, USA) ager, we colored the brain tissues for 1 min 40 s. We placed the stained tissues on slides and mounted them with DPX. We observed the NeuN-positive cells under an Olympus IX70 inverted microscope (Olympus Co., Tokyo, Japan). With Image J, we distinguished between neurons and background noise.

### 4.8. Behavior Test

To evaluate the neurological functioning of the rats in the TBI groups, we performed the modified neurological severity score (mNSS) and Morris water maze (MWM) tests, the results of which confirmed neurological deficit and cognitive dysfunction after TBI. For 7 days after TBI, we estimated the neurological severity score once per day with the mNSS test. This involved raising rats, placing them on the floor, a sensory test, and examining reflexes and abnormal movement after TBI. A higher score means that the brain had more severe damage. Each sham and TBI group comprised 4 and 6 rats, respectively. Next, we performed the MWM for one week after TBI. We estimated the escape time and distance in a water bath. MWM started on the 8th day after TBI and finished on 12th day after TBI. We placed the animals in 4 different starting zones, and we placed the escape platform near the first starting zone. From the 8th day after TBI, we measured the escape time and distance to target for 5 days. We considered a long tracking distance and escape time over 120 s as failure. We measured the tracking with smart video-tracking software 3.0 (Panlab, Carrer de l’Energia, Spain). We used 5 from each sham group and 10 from each TBI group.

### 4.9. Data Analysis

All data in this study were measured by Image J (National Institute of Health, Bethesda, MD, USA), and results are expressed as the mean ± SEM. To compare the vehicle and imipramine-treatment groups, we used the Mann–Whitney U test. A value of *p* < 0.05 indicated statistical significance. We analyzed all data with IBM SPSS statistics software.

## 5. Conclusions

Imipramine, an ASMase inhibitor, decreases ceramide formation, which leads to TBI-induced neuronal death. Thus, ASMase inhibition may be a promising therapeutic strategy to reduce TBI-induced hippocampal neuronal death.

## Data Availability

Not applicable.
